# Surgical Fear and Preference in Spine Disorders: A Prospective Study of Tomophobia and Tomophilia

**DOI:** 10.7759/cureus.115616

**Published:** 2026-09-01

**Authors:** Bharatkumar R Dave, Ajay Krishnan, Shivanand C Mayi, Ravi Ranjan Rai, Mirant B Dave, Mikeson Panthackel, Rushikesh B Shahade, Arjit Vashishtha, Yogenkumar Adodariya, Saurabh S Kulkarni, Kishan Panjwani

**Affiliations:** 1 Spine Surgery, Stavya Spine Hospital and Research Institute, Ahmedabad, IND; 2 Spine, Bhavnagar Institute Of Medical Sciences, Bhavnagar, IND; 3 Orthopedics, Seth Gordhandas Sunderdas Medical College (GSMC) and the King Edward Memorial (KEM) Hospital, Mumbai, IND; 4 Orthopedics, Mahatma Gandhi Medical College and Research Institute, Aurangabad, IND

**Keywords:** patient psychology, preoperative anxiety, spine surgery, tomophilia, tomophobia

## Abstract

Background

Treatment decisions in spine disorders are influenced by both clinico-radiological factors and psychological determinants. Tomophobia (fear of surgery) and tomophilia (positive inclination toward surgery) are emerging constructs that may significantly influence surgical decision-making, but remain insufficiently characterized. This study highlights the role of structured clinico-radiological correlation (CRC) combined with psychological assessment to improve accuracy of decision-making in spine care.

Methods and materials

This prospective observational study included 200 adult patients with cervical, thoracic, or lumbar spine disorders for whom both operative and non-operative management were clinically appropriate. The study was prospectively registered in the Clinical Trials Registry-India (CTRI No.: SSHRI/CS/NS/Tphobia-philia/BRD/88/12.25). Baseline demographic and clinical data were recorded. Pain and disability were assessed using a visual analog scale (VAS), Oswestry Disability Index (ODI), and Neck Disability Index (NDI). Psychological evaluation included Generalized Anxiety Disorder 7-item Scale (GAD-7), Pain Catastrophizing Scale (PCS), and a structured questionnaire assessing tomophobia and tomophilic inclination. All patients underwent CRC, and diagnostically uncertain or overlapping cases were further reviewed using a structured in-house multi-tier reassessment protocol. Associations between psychological traits and treatment preference were analyzed.

Results

Tomophobia was present in 62/200 patients (31.0%), while tomophilic inclination was observed in 138/200 patients (69.0%). Overall, 138/200 patients (69.0%) opted for surgical management, 51/200 patients (25.5%) chose conservative treatment, and 11/200 patients (5.5%) remained undecided following counselling. A significant association was observed between psychological disposition and treatment preference (p<0.001). Tomophobic patients predominantly selected conservative or undecided options, whereas tomophilic patients strongly preferred surgery. Tomophobia was associated with higher GAD-7 and PCS scores, while VAS and disability indices (ODI/NDI) were comparable across groups. CRC-guided in-house reassessment improved diagnostic clarity in selected overlap cases and refined treatment decisions.

Conclusions

Tomophobia and tomophilia represent important psychological determinants of treatment preference in spine disorders, operating independently of objective clinical severity. Patients with tomophobia demonstrate increased anxiety and catastrophizing tendencies, contributing to conservative or indecisive treatment choices, whereas tomophilic inclination is strongly associated with preference for operative management. The findings underscore the importance of integrating structured CRC with systematic psychological evaluation and a standardized in-house multidisciplinary review process to minimize diagnostic ambiguity, optimize patient selection, and ensure balanced shared decision-making in spine care.

## Introduction

Spine disorders, including lumbar disc herniation, spinal stenosis, and degenerative spondylolisthesis, are major contributors to pain, disability, and healthcare utilization worldwide. Low back pain affects approximately 60-80% of individuals during their lifetime and remains a leading cause of years lived with disability globally [1,2]. Although treatment decisions are traditionally guided by clinical findings, imaging severity, and neurological status, increasing evidence suggests that psychosocial factors significantly influence treatment acceptance and outcomes [3,4]. Accurate clinico-radiological correlation (CRC) remains fundamental in spine decision-making, particularly in patients with overlapping or disproportionate symptom profiles.

Psychological constructs such as anxiety, fear-avoidance beliefs, and pain catastrophizing are well-established determinants of pain perception, functional limitation, and postoperative recovery in spine disorders. Higher psychological distress has been associated with increased pain intensity, delayed recovery, and reduced treatment satisfaction, and may also affect adherence to recommended management strategies and patient expectations [5-7]. These factors collectively influence both conservative and surgical decision-making pathways [8].

Among these constructs, tomophobia, defined as excessive fear of surgical intervention, represents an under-recognized barrier to timely operative care. It may lead to refusal or delay of indicated surgery, even in cases with clear clinical need [9,10]. Conversely, tomophilia, defined as a positive inclination toward surgical intervention, may predispose patients toward operative preference even when conservative management remains appropriate, representing an underexplored behavioral dimension in surgical decision-making [11].

Shared decision-making in spine care requires integration of clinical evidence with patient values, expectations, and risk perception. Socioeconomic status, educational background, and health literacy further shape patient attitudes toward surgery, particularly in resource-variable healthcare settings such as India, where variability in access and awareness may influence treatment preferences [12,13]. Despite growing recognition of psychological influences in spine care, structured evaluation of surgery-related fear and positive surgical inclination remains limited. Most prior studies have focused on general surgical anxiety or fear-avoidance behavior, without specifically examining dual constructs such as tomophobia and tomophilia in spine populations. Furthermore, prospective data correlating these constructs with treatment preference and psychological profiling remain scarce [14,15].

Accordingly, this prospective observational study aimed to estimate the prevalence of tomophobia in patients with spine disorders where both operative and non-operative options were clinically viable. The study further evaluated the association between surgery-related psychological dispositions and treatment preference, while assessing related demographic, clinical, anxiety, and pain catastrophizing factors. In addition, the study explored the role of positive surgical inclination (tomophilia) as a complementary behavioral construct influencing decision-making in spine care.

## Materials and methods

Study design and setting

This was a hospital-based prospective observational study conducted at Stavya Spine Hospital and Research Institute, Ahmedabad, India, a tertiary referral spine care centre. A total of 200 adult patients with cervical, thoracic, or lumbar spine disorders were prospectively enrolled. The study was conducted over a period of four months from Jan to April 2026. Patients were included when both operative and non-operative treatment options were clinically reasonable, allowing assessment of treatment preference patterns and surgery-related psychological dispositions. Ethical approval was obtained with Protocol No.: SSHRI/CS/NS/Tphobia-philia/BRD/96/12.25, and CTRI No.: SSHRI/CS/NS/Tphobia-philia/BRD/88/12.25, and written informed consent was obtained from all participants. The study was conducted in accordance with the Declaration of Helsinki.

Study population

Patients aged ≥18 years with clinically and radiologically confirmed cervical, thoracic, or lumbar spine disorders were included. Only those in whom both operative and non-operative management were considered appropriate based on initial evaluation were enrolled. Patients presenting with emergency conditions such as cauda equina syndrome, acute spinal trauma, or rapidly progressive neurological deficit were excluded. Additional exclusion criteria included cognitive impairment preventing questionnaire completion, prior spine surgery within the preceding six months, and refusal to participate. Consecutive eligible patients were enrolled during the study period.

Clinical assessment and clinico-radiological correlation (CRC)

Baseline demographic variables including age, sex, education level, and occupation were recorded. Clinical evaluation included detailed history, neurological examination, and provisional diagnosis based on standard spine assessment protocols. A structured CRC approach was applied in all patients to ensure alignment between symptoms, examination findings, and imaging features before final treatment recommendation. In cases of diagnostic ambiguity or symptom overlap, a standardized three-tier in-house evaluation system was implemented. This consisted of sequential assessment by a senior resident, detailed evaluation by the treating consultant, and final confirmation by the chief spine surgeon to ensure diagnostic consistency and minimize cognitive bias. In selected cases with overlapping musculoskeletal conditions (e.g., knee-spine syndrome), adjunctive diagnostic interventions such as intra-articular knee injections with lignocaine were used to identify the dominant pain generator and refine treatment sequencing.

Psychological and functional assessment

Functional disability was assessed using the Oswestry Disability Index (ODI) for lumbar spine disorders [7] and the Neck Disability Index (NDI) for cervical spine disorders [8]. Pain intensity was measured using the Visual Analog Scale (VAS; 0-10) [16].

Psychological assessment was performed using validated instruments, including the Generalized Anxiety Disorder-7 (GAD-7) questionnaire [10] and the Pain Catastrophizing Scale (PCS) [12].

Additionally, a study-specific questionnaire was administered to evaluate surgery-related psychological attitudes (appendix). The questionnaire was developed through multidisciplinary expert consensus involving spine surgeons and psychologists. Face and content validity were assessed prior to pilot testing. Internal consistency was acceptable (Cronbach’s alpha = 0.82). The instrument assessed fear of surgery, anesthesia-related concerns, perceived risk of neurological worsening or postoperative disability, fear of delayed surgery consequences (including worsening disability with non-intervention), and perceived benefits of surgical treatment. Responses were recorded using Likert-scale grading.

Operational classification of tomophobia and tomophilia

Tomophobia and tomophilia were classified using prespecified responses to the study-specific psychological questionnaire, completed before documentation of the patient's final treatment preference. Tomophobia was defined by a predominance of fear-oriented responses regarding surgery, anesthesia, anticipated complications, postoperative disability, and perceived surgical risks, whereas tomophilia was defined by a predominance of positive responses regarding the perceived benefits of surgery, confidence in surgical outcomes, and preference for definitive operative treatment. Treatment preference was not used as a criterion for psychological group assignment.

Treatment preference assessment

All patients underwent standardized counselling by spine surgeons, including detailed explanation of diagnosis, available treatment options, expected outcomes, and risks and benefits of both surgical and non-surgical management. Patients were also informed about the potential consequences of delayed surgery when clinically indicated, as part of routine informed consent discussion. Educational aids, including anatomical models and imaging-based visual tools, were used to improve understanding. Treatment preference was recorded at baseline after completion of psychological assessment and counselling and categorized as surgical, conservative, or undecided.

Statistical analysis

Statistical analysis was performed using IBM SPSS Statistics for Windows, Version 26 (Released 2019; IBM Corp., Armonk, New York, United States). Continuous variables were expressed as mean ± standard deviation, and categorical variables as frequencies and percentages. Normality was assessed prior to parametric testing. Associations between categorical variables were evaluated using the chi-square test or Fisher’s exact test where appropriate. Independent sample t-tests were used for comparison of continuous variables between groups. All tests were two-tailed, and a p-value <0.05 was considered statistically significant.

## Results

Baseline demographic and socioeconomic characteristics

A total of 200 patients were included in this prospective observational study. The mean age of the cohort was 56.96 ± 13.53 years, representing a predominantly middle-aged and elderly population. Female subjects accounted for 110 patients (55.0%), while male subjects accounted for 90 patients (45.0%). Most participants had undergraduate education (n=134, 67.0%), followed by graduates (n=50, 25.0%), with smaller proportions of uneducated (n=12, 6.0%) and postgraduate (n=4, 2.0%) individuals. Housewives constituted the largest occupational subgroup (n=109, 54.5%), followed by retired individuals (n=38, 19.0%) (Table 1).

**Table 1:** Baseline demographic and socioeconomic characteristics of the study population (n=200) Data are represented as mean ± SD for continuous variables (Age) and as n (%) for categorical variables.

Variable	Category	Number (n) and percentage (%)/Mean ± SD
Age (years)	Mean ± SD	56.96 ± 13.53
Sex	Male	90 (45.0%)
	Female	110 (55.0%)
Education	Under Graduate	134 (67.0%)
	Graduate	50 (25.0%)
	Uneducated	12 (6.0%)
	Post Graduate	4 (2.0%)
Occupation	Housewife	109 (54.5%)
	Retired	38 (19.0%)
	Job	16 (8.0%)
	Business	14 (7.0%)
	Farmer	7 (3.5%)
	Shopkeeper	4 (2.0%)
	Worker	2 (1.0%)
	Others	10 (5.0%)

Clinical characteristics

Degenerative lumbar spine disorders constituted the predominant clinical presentations. The most frequently observed diagnosis was L4-L5 spinal stenosis, followed by mechanical low back pain and L4-L5 spondylolisthesis. Additional diagnoses included lumbar disc prolapse, muscle strain, and postural pain syndromes, reflecting a heterogeneous spectrum of degenerative and mechanical spinal conditions (Table 2).

**Table 2:** Distribution of primary clinical diagnoses among the study population (n=200) Data are represented as n (%). Degenerative lumbar spine disorders constituted the predominant clinical presentations. Knee-Spine syndrome, mechanical low back pain, postural pain syndromes, and muscle strain collectively represented the largest subgroup categorized under other diagnoses.

Diagnosis	Number; n (%)
L4–L5 Spinal Stenosis	23 (11.5%)
L4–L5 Spondylolisthesis	15 (7.5%)
L5–S1 Spondylolisthesis	10 (5.0%)
L5–S1 Prolapsed Intervertebral Disc	8 (4.0%)
L4–L5 Prolapsed Intervertebral Disc	6 (3.0%)
Other Diagnoses i.e. Knee-Spine syndrome, Mechanical back pain, postural pain, Muscle strain	138 (69.0%)

Psychological profile and treatment preference

Patients were stratified into two mutually exclusive psychological groups based on dominant response patterns: tomophobic (n=62, 31.0%) and tomophilic (n=138, 69.0%). At baseline, 138 patients (69.0%) preferred surgical management, 51 patients (25.5%) opted for conservative treatment, and 11 patients (5.5%) remained undecided following standardized counselling. A statistically significant association was observed between psychological disposition and treatment preference (p<0.001). Patients classified as tomophobic predominantly opted for conservative management or remained undecided, whereas patients classified as tomophilic demonstrated a near-universal preference for surgical intervention, indicating a strong influence of psychological disposition on treatment decision-making independent of baseline clinical severity (Table 3).

**Table 3:** Psychological group and treatment preference Distribution of treatment preference according to psychological disposition. Data are represented as n (%). A Pearson Chi-Square test revealed a statistically significant association between psychological group and treatment preference (χ^2^ = 200.02, p<0.001).

Psychological Group	Conservative; n (%)	Surgery; n (%)	Undecided; n (%)
Tomophobic (n=62)	51 (82.3)	0 (0)	11 (17.7)
Tomophilic (n=138)	0 (0)	138 (100)	0 (0)

Interestingly, these findings suggest that positive expectations regarding operative intervention may influence treatment preference independently of objective surgical pathology. These conditions included mechanical low back pain, muscle strain, and postural pain syndromes. Surgical preference in these patients was observed despite the absence of classical compressive or instability-related surgical pathology, suggesting that psychological predisposition toward operative intervention may influence treatment expectations independent of objective surgical necessity (Figure 1).

**Figure 1:**
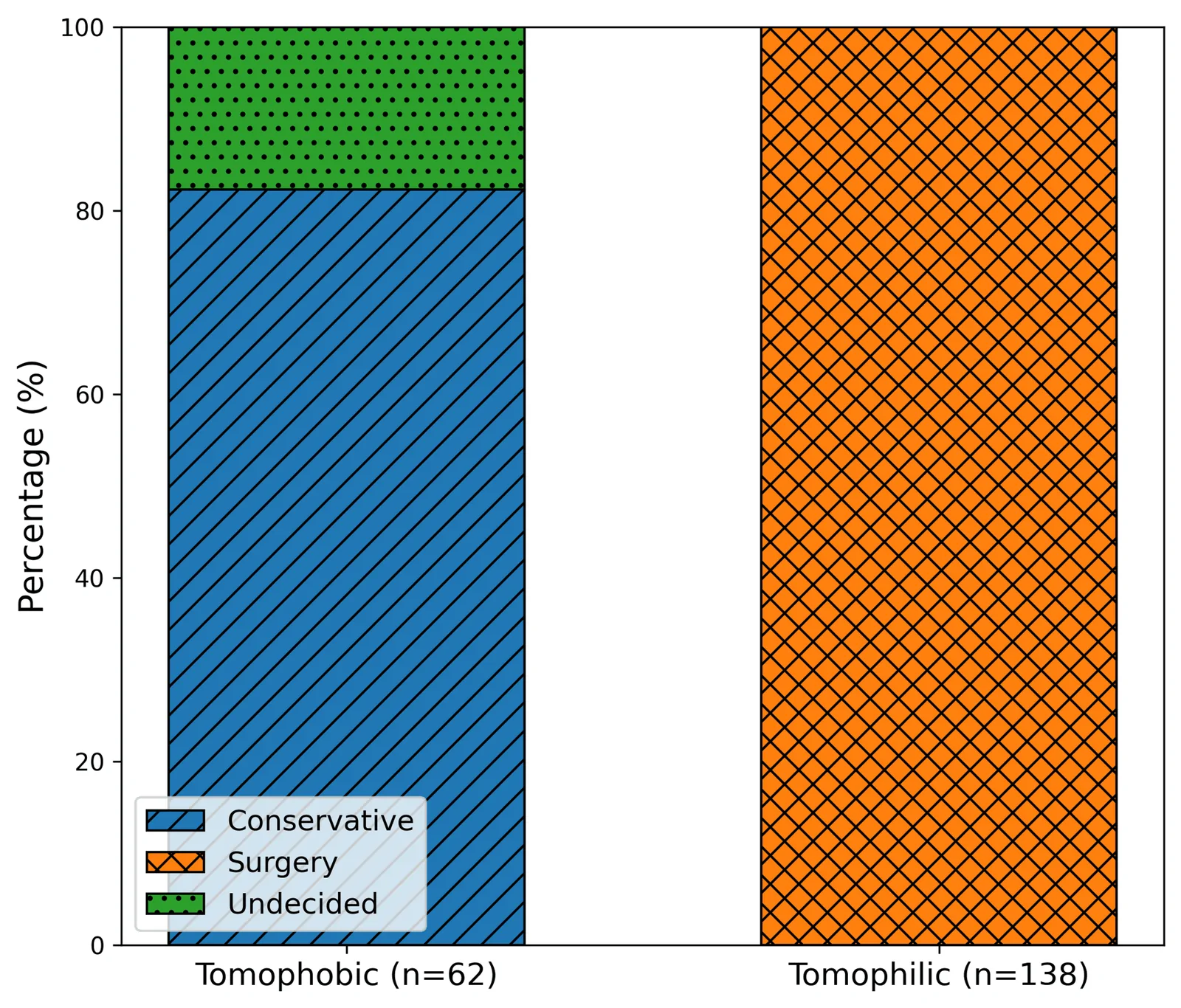
Proportional distribution of treatment preferences across psychological traits is illustrated using a 100% stacked bar chart Data are represented as percentages (%). A p-value of <0.05 was considered statistically significant.

Tomophilic group: conservative-type diagnoses and structured in-house reassessment

Among the 138 patients classified as tomophilic, 31 (22.5%) represented a clinically important subgroup demonstrating strong surgical preference despite conservative-type diagnoses.These included Knee-Spine syndrome, mechanical low back pain, and postural pain syndromes. This subgroup demonstrated moderate symptom burden, with a mean VAS score of 5.94 ± 1.12 and mean ODI of 38.42 ± 10.84, although radiological assessment did not demonstrate surgically significant compressive or instability-related pathology (Table 4).

**Table 4:** Conservative-type diagnoses in tomophilic patients with initial surgical preference Subgroup of tomophilic patients demonstrating strong preference for surgical intervention despite absence of surgically significant pathology. Structured three-check in-house evaluation led to reassessment and appropriate redirection toward conservative or staged management. Data are represented as n (%) for categorical variables and as mean ± SD for continuous variables (visual analog scale (VAS), Oswestry Disability Index (ODI)).

Diagnosis	n (%)	Mean VAS	Mean ODI (%)
Knee-Spine syndrome	15 (48.4)	6.1 ± 1.0	39.5 ± 11.2
Mechanical Low Back Pain	9 (29.0)	5.8 ± 1.3	36.7 ± 9.8
Postural Pain Syndrome	7 (22.6)	5.7 ± 1.1	37.2 ± 10.1
Total	31 (100)	5.94 ± 1.12	38.42 ± 10.84

All such cases underwent a structured three-check in-house evaluation system, comprising sequential assessment by a senior resident, detailed evaluation by the primary consultant, and final confirmation by the chief spine surgeon. In cases with overlapping symptomatology, particularly knee-spine overlap syndrome, additional diagnostic local lignocaine intrarticular knee injections were utilized to identify the dominant pain generator and to assist in treatment sequencing decisions. Following this structured in-house reassessment, patients were redirected toward conservative or staged non-operative management, and in a limited subset, diagnostic reclassification was performed after multidisciplinary review.

Clinical scenarios demonstrating positive surgical inclination and diagnostic complexity

Approximately 15 patients (7.5%) presented with coexisting spinal and non-spinal pathologies, including knee osteoarthritis and/or vascular claudication in association with lumbar spinal stenosis (Table 5).

**Table 5:** Coexisting conditions and final treatment selection Patients with overlapping spinal and non-spinal pathologies evaluated using a structured three-check in-house system. Multidisciplinary reassessment facilitated further clinical clarification and modification of treatment selection in selected cases. Data are represented as n (%).

Condition	Total (n)	Spine Surgery; n (%)	Non-Spinal Intervention; n (%)
Knee Osteoarthritis + Lumbar Stenosis	9	6 (66.7)	3 (33.3)
Vascular Claudication + Lumbar Stenosis	4	3 (75.0)	1 (25.0)
Knee Osteoarthritis + Vascular Disease + Lumbar Stenosis	2	1 (50.0)	1 (50.0)
Total	15	10 (66.7)	5 (33.3)

These cases represented diagnostically complex scenarios requiring structured multidisciplinary evaluation. Each case underwent a standardized three-tier in-house assessment system consisting of resident evaluation, consultant review, and final confirmation by the chief spine surgeon, supported by comprehensive clinical and radiological correlation to determine the primary pain generator.

In cases of diagnostic ambiguity, second in-house opinion and targeted diagnostic knee injections were employed in knee-spine overlap syndrome to improve diagnostic precision and guide appropriate treatment sequencing. In three patients, this structured evaluation resulted in revision of the initial diagnostic impression and modification of the treatment plan, thereby avoiding potentially unnecessary spinal surgical intervention.

Association of psychological group with anxiety severity

A statistically significant association was observed between psychological grouping and anxiety severity (p<0.001). Patients in the tomophobic group demonstrated higher levels of anxiety, whereas those in the tomophilic group predominantly exhibited mild anxiety levels.

Patients with tomophilia predominantly demonstrated mild anxiety (92.8%), with only one patient exhibiting moderate anxiety, whereas patients with tomophobia most commonly exhibited moderate (75.8%) and severe anxiety (22.6%). These findings indicate a strong relationship between surgery-related fear and higher anxiety severity (Table 6).

**Table 6:** Distribution of anxiety scores in patients based on Generalised anxiety disorder (GAD) scores among those with tomophilia and tomophobia Data are represented as n (%). A Pearson Chi-Square test indicated a statistically significant difference in anxiety severity between groups (χ^2^ =190.73, p<0.001).

Variable	Minimal; n (%)	Mild; n (%)	Moderate; n (%)	Severe; n (%)	Total
Tomophobia Present	0 (0%)	1 (1.6%)	47 (75.8%)	14 (22.6%)	62
Tomophilia Present	9 (6.5%)	128 (92.8%)	1 (0.7%)	0 (0%)	138

Comparison of clinical and psychological parameters

No statistically significant differences were observed between groups in VAS, ODI, or NDI scores (p>0.05), indicating comparable baseline clinical severity. However, PCS and GAD-7 scores were significantly higher in the tomophobic group (p<0.001), suggesting greater psychological distress despite similar clinical and radiological disease burden. The exact p-values are reported in Table 7.

**Table 7:** Comparison of clinical, functional, and psychological parameters between patients with tomophobia and tomophilia Pain intensity (VAS) and disability indices (ODI and NDI) were comparable between groups; however, significantly higher PCS and GAD-7 scores were observed in patients with tomophobia (p<0.001).Values are expressed as mean ± standard deviation (SD). VAS: Visual Analog Scale; ODI: Oswestry Disability Index; NDI: Neck Disability Index; PCS: Pain Catastrophizing Scale; GAD-7: Generalized Anxiety Disorder-7 scale. A p-value of <0.05 was considered statistically significant. *Statistically significant difference between groups.

Parameter	Tomophobia present (n=62)	Tomophilia present (n=138)	Test statistic (t-value)	p-value
VAS Score	5.82 ± 1.24	5.81 ± 1.36	t = 0.05	0.960
ODI (%)	42.76 ± 12.92	43.34 ± 14.14	t = -0.28	0.780
NDI (%)	44.57 ± 18.32	42.13 ± 16.92	t = 0.92	0.359
PCS Score	20.65 ± 2.82	19.22 ± 1.84	t = 4.28	<0.001*
GAD-7 score	13.10 ± 2.15	12.33 ± 1.88	t = 2.56	0.011*

Figure 2 illustrates the comparative distribution of mean VAS, ODI/NDI, PCS, and GAD-7 scores between the tomophobic and tomophilic groups.

**Figure 2:**
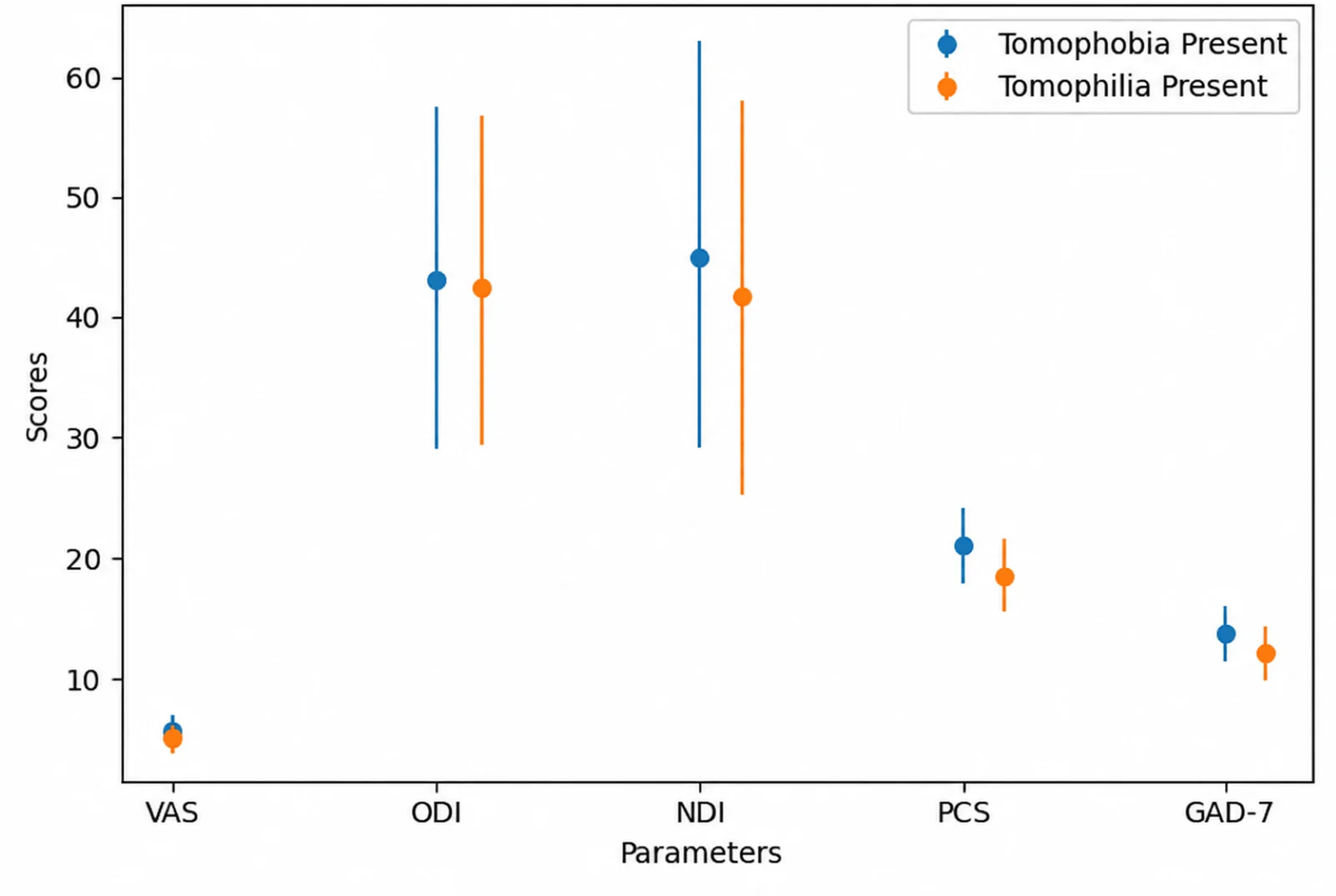
Dot-and-error bar plots demonstrating mean ± SD values of VAS, ODI/NDI, PCS, and GAD-7 scores among patients with and without tomophobia Data are represented as mean ± SD. VAS: Visual Analog Scale; ODI: Oswestry Disability Index; NDI: Neck Disability Index; PCS: Pain Catastrophizing Scale; GAD-7: Generalized Anxiety Disorder-7 scale.

While pain intensity and functional disability measures were comparable between the groups, patients with tomophobia demonstrated higher levels of pain catastrophizing and anxiety. 

## Discussion

Treatment decision-making in degenerative spine disorders is increasingly recognized as a complex interaction between structural pathology and patient-level psychological factors. In clinical spine practice, operative and non-operative pathways often coexist as valid options, making patient perception, risk appraisal, and cognitive-emotional bias important determinants of final management selection. In this context, surgery-related psychological constructs such as tomophobia and tomophilia represent two contrasting but clinically relevant behavioral dimensions influencing treatment preference. Tomophobia reflects avoidance-driven fear of surgical intervention, whereas tomophilia represents a positive, approach-oriented inclination toward operative management. In the present prospective cohort, these constructs were systematically evaluated alongside validated clinical and psychological parameters to assess their impact on treatment decision-making in degenerative spine disorders [1-3]. However, the conceptual overlap between positive surgical inclination and final treatment preference remains an important methodological consideration requiring further psychometric validation.

In the present study, tomophobia was identified in 31.0% of patients, which is consistent with previously reported prevalence ranges of preoperative surgical anxiety in elective orthopedic and spine populations (approximately 20-40%) [1-3]. A statistically significant association was observed between psychological disposition and treatment preference (p<0.001), with tomophobic patients predominantly opting for conservative management or remaining undecided, while tomophilic patients demonstrated a strong preference for surgical intervention. Importantly, this association persisted despite comparable clinical severity across groups, as reflected by similar VAS, ODI, and NDI scores [4-6]. This dissociation between symptom burden and treatment preference reinforces the concept that structural severity alone is insufficient to explain surgical decision-making, and that psychological constructs exert an independent influence on treatment pathways [7].

From a mechanistic standpoint, tomophobia aligns with established fear-avoidance and cognitive threat appraisal models, wherein catastrophic interpretation of pain, procedural fear, and anticipated postoperative harm contribute to avoidance behavior. In the present cohort, tomophobic patients demonstrated significantly higher GAD-7 and PCS scores, indicating a measurable clustering of anxiety and maladaptive pain cognition [8,9]. This is consistent with prior evidence demonstrating that elevated preoperative anxiety is associated with increased pain perception, heightened perioperative distress, and poorer functional recovery following spine surgery [10-12]. Similarly, pain catastrophizing has been shown to amplify nociceptive processing, reduce coping capacity, and negatively affect rehabilitation outcomes, thereby reinforcing conservative preference even in clinically suitable surgical candidates [9,13,14]. Identification of surgery-related psychological disposition may help prevent both delayed necessary surgery and potentially avoidable operative intervention.

In contrast, tomophilia represents a distinct psychological construct characterized by an active preference for surgical intervention rather than absence of fear alone. In this study, tomophilic patients demonstrated a disproportionately high inclination toward operative management, including in cases with moderate symptom burden and conservative-type pathology. This suggests that tomophilia may be driven by positive outcome expectancy, perceived superiority of surgical correction, sociocultural reinforcement of operative solutions, and prior exposure to favorable surgical outcomes. Unlike tomophobia, which reflects avoidance-based cognition, tomophilia reflects approach-oriented decision bias that may potentially lead to overestimation of surgical benefit in borderline or non-indicated clinical scenarios [15].

A clinically important finding in this study was the presence of diagnostic incongruence in a subset of tomophilic patients, where strong surgical demand coexisted with conditions typically managed conservatively, including Knee-Spine syndrome, mechanical low back pain and postural pain syndromes. These patients demonstrated moderate symptom burden (mean VAS approximately 5.9; ODI approximately 38%) but lacked radiological evidence of surgically actionable pathology. To address this mismatch between patient expectation and objective clinical indication, a structured in-house three-tier evaluation system was implemented, consisting of sequential assessment by a senior resident, detailed evaluation by the primary consultant, and final confirmation by the chief spine surgeon. This stepwise “three-check” system served as a cognitive de-biasing framework to ensure adherence to evidence-based surgical indications and reduce the risk of unnecessary intervention.

In selected cases with diagnostic ambiguity, particularly in knee-spine overlap syndrome or combined musculoskeletal and vascular symptomatology, targeted diagnostic adjuncts such as intra-articular knee injections were used to identify the dominant pain generator. This approach allowed functional differentiation between spinal and non-spinal sources of pain, which is often challenging in degenerative multimorbidity. In a small subset of patients, this structured reassessment led to revision of the initial diagnosis and modification of treatment strategy, thereby preventing potentially unwarranted spine surgery and improving diagnostic precision.

The cohort with coexisting spinal and non-spinal pathologies further highlighted the complexity of symptom attribution in degenerative disease. Patients presenting with combinations of knee osteoarthritis, vascular claudication, and lumbar spinal stenosis required systematic hierarchical evaluation to determine the primary pain source. The integration of the three-tier in-house evaluation system with second-opinion review and selected diagnostic interventions facilitated further clinical clarification and appropriate sequencing of interventions. In a minority of cases, this process altered the primary treatment target, underscoring the importance of structured multidisciplinary reassessment in avoiding diagnostic anchoring and overtreatment.

Collectively, these findings emphasize that spine surgical decision-making is not solely dependent on anatomical pathology but is significantly influenced by quantifiable psychological constructs. The coexistence of tomophobia and tomophilia suggests a bidirectional psychological spectrum influencing surgical decision behavior, where both avoidance and over-acceptance of surgery may deviate from optimal clinical indication thresholds. This has direct implications for shared decision-making frameworks and highlights the need for routine incorporation of validated psychological screening tools such as GAD-7 and PCS into preoperative assessment protocols [10,13,17]. From a systems perspective, the structured in-house three-tier evaluation model represents a reproducible clinical governance framework for minimizing diagnostic error in complex spine presentations. This is particularly relevant in resource-variable settings where overlapping musculoskeletal, vascular, and degenerative conditions are common, and where patient-driven surgical preference may not always correlate with objective surgical necessity.

This study has several limitations. Being a single-center study, external generalizability is limited. The study-specific operational definitions of tomophobia and tomophilia, although internally consistent, require external validation in larger multicentric cohorts. The study-specific questionnaire was internally validated but lacked external psychometric validation. Additionally, cross-sectional assessment limits inference regarding temporal stability of psychological constructs and their long-term impact on treatment outcomes.

## Conclusions

Psychological disposition significantly influences treatment preference in degenerative spine disorders. Tomophobia was associated with higher anxiety and PCS and a tendency toward conservative management or indecision. In contrast, tomophilic inclination showed a strong association with preference for surgical intervention, even in cases without proportional radiological severity. This indicates that treatment decisions are not solely driven by clinical severity or imaging findings but are also modulated by psychological factors.

These findings highlight the need to integrate CRC with structured psychological assessment to ensure accurate decision-making. A standardized in-house three-tier evaluation system - consisting of sequential assessment by a senior resident, consultant review, and final chief surgeon confirmation - was used to minimize diagnostic bias. In selected cases, adjuncts such as diagnostic knee injections aided identification of the primary pain generator in overlap syndromes. Systematic evaluation of both tomophobia and tomophilia within this CRC-based framework is essential for completing the diagnostic process and ensuring appropriate treatment alignment.

## Appendices

**Table 8:** Study-specific questionnaire administered to evaluate surgery-related psychological attitudes This structured evaluation tool was administered to all enrolled patients to capture baseline demographic and clinical data, verify inclusion/exclusion criteria, and document objective functional scores (VAS, ODI/NDI, PCS, GAD-7). Furthermore, it identifies and ranks the specific patient-reported priorities driving tomophobic (surgery-avoidant) or tomophilic (surgery-inclined) treatment preferences. PhD: Doctor of Philosophy; VAS: Visual Analog Scale; ODI: Oswestry Disability Index; NDI: Neck Disability Index; PCS: Pain Catastrophizing Scale; GAD-7: Generalized Anxiety Disorder-7; MRI: Magnetic Resonance Imaging; CT: Computed Tomography.

Age	
Sex	Male
	Female
Occupation	
Education	Uneducated
	Under-graduate
	Graduate
	Post-graduate
	PhD
Contact Number	
State & City	
Provisional Diagnosis	
Duration of symptoms	
Previous spine treatments- please tick the right answer	Medications
	Physiotherapy
	Injections
	Previous spine surgery
Co-morbidities	
Criteria	
Inclusion criteria	Adults aged ≥18 years
	Diagnosed with a cervical, thoracic, or lumbar spine condition for which surgery is an option
Exclusion criteria	Emergency or urgent surgery (e.g., cauda equina, acute trauma)
	Spine surgery performed within the last 6 months
“WHY” TOMOPHOBIA Prioritize ANY 3 fears from greatest to least concern	1. Fear of paralysis
	2. Poor outcomes or unsuccessful surgery
	3. Fear of Implants
	4. Fear of anesthesia
	5. Fear of risks related to co-morbidities (like diabetes, hypertension etc).
	6. Fear of age
	7. Fear of after-surgery restriction
“WHY” TOMOPHILIA Prioritize ANY 3 desires from greatest to least concern.	1. Desire of fitness (Relief from disability)
	2. Desire of unused claims/Company benefits
	3. Desire of pain relief
	4. Desire of freedom from disease
	5. Desire of quick relief
	6. Desire of prevention development of disease
Type of Spinoxiety	Type 1 (Self-induced)
	Type 2 (Secondary induced)
Baseline assessments	VAS Pain Score
	ODI/NDI Score
	Pain Catastrophizing Scale (PCS)
	GAD-7 Anxiety Severity
	Neurological exam
Patient’s preference	Surgery
	Conservative
	Undecided
